# Services for women’s sexual and reproductive health in India: an analysis of treatment-seeking for symptoms of reproductive tract infections in a nationally representative survey

**DOI:** 10.1186/s12905-020-01024-3

**Published:** 2020-07-28

**Authors:** Shikha Bhasin, Ankita Shukla, Sapna Desai

**Affiliations:** Population Council, Zone 5A, Habitat Centre, New Delhi, India

**Keywords:** Reproductive tract infections; gynaecological morbidity, Treatment seeking, India, women’s health

## Abstract

**Background:**

Women’s health policy in India has had a longstanding focus on maternal health and family planning. Recent policy highlights the importance of expanding women’s access to a broader range of sexual and reproductive health services. However, there has been very limited analysis of national survey data to examine the current status of treatment utilisation, variation across states and progress over time.

**Methods:**

This paper examines women’s treatment patterns for reproductive tract infections in India, based on data collected in the National Family Health Survey, a cross-sectional, nationally representative household survey conducted between 2015-16. The survey covered 699,686 women between the ages 15 and 49, of which 91,818 ever sexually active women responded to questions related to symptoms of reproductive tract infections. We estimate prevalence of reported symptoms and treatment-seeking, describe regional variation and utilise multivariable logistic regression to identify factors associated with women’s treatment-seeking patterns.

**Results:**

Thirty-nine percent of women who reported symptoms of reproductive tract infections sought any advice or treatment. Women’s reported treatment-seeking in India has not changed since the last national survey a decade earlier. Reported symptoms and treatment-seeking varied widely across India, ranging from 64% in Punjab to 8% in Nagaland, with no clear regional pattern that emerged. Seventeen percent of symptomatic women sought services in the public sector, an improvement from 11% in 2005–06. Twenty-two percent utilised the private sector, with wide variation by states. National-level multivariable logistic regression indicated that treatment-seeking was associated with age, higher education, higher household wealth and having been employed in the past year. Women in the 25–35 age group had higher odds (aOR1.27; 95% CI: 1.10,1.50) of seeking treatment compared to both younger (15–19 years) and older (35 years and above) women, along with women with more than eight years of schooling (aOR: 1.23; 95% CI: 1.05,1.44) and from richer wealth quintiles (aOR: 1.53; 95% CI: 1.35,1.83).

**Conclusion:**

Women’s use of services for reproductive tract infections remains a challenge in most parts of India. Our findings highlight the need to address barriers to seeking care and to improve measurement of gynaecological ailments in national surveys.

## Background

Expanding priorities for women’s health beyond family planning and maternal health has been an important achievement of global policy advocacy in the past twenty-five years [1, 2]. Policies and international commitments have progressed from population control to seeking to ensure sexual and reproductive health and rights within Universal Health Coverage (UHC) – albeit with challenges in achieving a comprehensive women’s health approach through the life cycle [1, 3]. In India, policies since the 2000s and the recent National Health Policy (2017) have supported the expansion of women’s health beyond maternal health to include treatment for reproductive tract infections (RTIs), cervical cancer screening and non-communicable diseases [4]. Most recently, the 2018 India Strategy for Women, Adolescents and Child Health (I-WACH) builds on these policies to articulate a life-course approach to women’s health that encompasses prevention, promotion, treatment and social determinants of health [5]. As India advances on the path towards UHC, it is critical to reflect on whether shifts in policy priorities to expand women’s health beyond maternal health and family planning have translated into increased service utilisation by women [4].

Population-based surveys in India provide an opportunity to examine progress over time and across states for some indicators of women’s health. The country’s major health surveys, the National Family Health Survey (NFHS) and sub-national District Level Household Survey (DLHS), focus primarily on maternal and child health and family planning, with more recent inclusion of intimate partner violence and risk factors for non-communicable disease [6]. The DLHS collects data on gynaecological morbidity, specifically symptoms of menstrual disorders and reproductive tract infections (RTIs), and the NFHS only collects evidence on the latter. While tracking women’s vulnerability to cardiovascular disease and cancer is a recent initiative, treatment for RTIs has been a longstanding measurement and policy priority.

Reproductive tract infections, which commonly may be undiagnosed or untreated, can lead to complications such as pelvic inflammatory disease, chronic pelvic pain and infertility, adverse pregnancy outcomes, as well as increased risk of HIV transmission [7]. National surveys in India define symptoms of RTIs as abnormal genital discharge, ulcers, sores or other ailments due to sexual contact, thus focussing on a subset of sexually transmitted infections (STIs). They do not include other symptoms of infections, such as burning urination or pelvic pain. In 1998–9, the NFHS-2 estimated that 35% of ever sexually active women who reported symptoms of RTIs had sought advice or treatment [8]. The following decade, the DLHS-3 (2008–9), a sub-nationally representative survey conducted amongst ever-married women, reported that 40% of symptomatic women sought treatment for RTIs and 12% reported menstrual disorders [9].

A systematic review of seventeen community-based studies on treatment for RTIs and STIs in India, across rural and urban populations in most states except Kerala and the North East, estimated that between 16 to 55% of women with symptoms sought treatment [10]. Community-based research has also highlighted variation in self-reported symptoms and treatment across geographic context and by women’s own perceptions of morbidity. Different methodological approaches, such as studies that use self-reported symptoms compared to those that employ clinical diagnosis, render comparison difficult across settings [10–14]. Nonetheless, community-based research has consistently identified barriers to treatment seeking for RTIs, and the critical importance of expanding women’s access to appropriate, accessible treatment [14–18].

Since the last round of the NFHS in 2005–6, India’s National Health Mission has introduced a range of measures to improve women’s utilisation of maternal and child health services. The most recent NFHS round (2015–16) provides an opportunity to review women’s treatment patterns for RTIs, in light of progress in maternal health and recent policy commitments to expand SRH services. This paper utilises nationally representative data to examine: (i) the current status of women’s treatment-seeking for symptoms of RTIs; (ii) state-level variation; and (iii) correlates of seeking treatment. We also identify gaps in the measurement of women’s gynaecological morbidity in national surveys to improve monitoring of, and action for, women’s health in India.

## Methods

The study draws from the fourth round of the National Family Health Survey (NFHS-4), a nationally representative, cross-sectional, household sample survey conducted in all states and union territories of India [19]. The NFHS provides estimates on population, health and nutrition as reported by adults aged 15–49. The NFHS-4 survey adopted a stratified two-stage sampling design, utilising the 2011 census as the sampling frame for the selection of primary sampling units (PSUs). PSUs, villages in rural areas and census enumeration blocks in urban areas, were selected using probability proportional to size sampling. Households were selected through systematic random selection of households within each PSU [20].

The Woman’s Questionnaire collected information from women aged 15–49 on: reproductive and sexual health including contraception, maternal health and gender-based violence; empowerment-related issues such as decision-making and mobility; and non-communicable diseases. State-level modules included three questions about RTIs for women who reported being sexually active, irrespective of their marital status: experience of ailments due to sexual contact; bad-smelling abnormal genital discharge; and the presence of genital sores or ulcers, all within the twelve- month period before the survey.

Women who reported at least one symptom were asked a follow-up question about whether they sought treatment/advice and a multiple response question on the facilities where treatment was sought. Two binary outcome variables were constructed based on whether women reported at least one of the symptoms of RTIs and whether they sought any treatment/advice. Data regarding treatment/advice for reported symptoms were collected according to type of facility visited by the respondent, categorized as public, private, or others. Public included government hospital, government AYUSH doctor, government health centre, family planning clinic, mobile clinic, govt. fieldworker, school-based clinic or other public facilities. Private included private hospital/clinic/doctor, private AYUSH doctor, pharmacy, private mobile clinic, private health worker and other private facilities. Other facilities included non-government organisations, home treatment, correctional facility and other facilities.

We identified independent variables for crude analyses based on a review of the literature and availability of data in the NFHS-4. These included: woman’s age (15–25, 25–35 and more than 35 years); years of schooling (none, 1–8 years and more than 8 years); religion (Hindu, Muslim, and others); caste (scheduled tribe/caste (ST/SC), other backward caste (OBC) and others); residence (urban and rural); household wealth index (a composite score based on household assets categorized into three categories: poor, middle and rich); marital status (unmarried and married); engaged in work in the last year (yes/no); if women considered distance to a health facility a problem (yes/no); freedom of mobility (no mobility at all and mobility for at least one of the following-to go market, health facility or outside village); role in decision-making (none at all or for at least one of: health care, large household purchases and daily needs); and exposure to intimate partner violence in the home (yes/no).

The survey’s state modules collected information on symptoms of RTIs from 91,818 women who reported being sexually active. We estimated prevalence and treatment-seeking based on these self-reported responses, presented with 95% confidence intervals and geographic distribution. We calculated unadjusted odds ratios to estimate associations for variables identified from the literature. The multivariable regression model to estimate adjusted odds ratios included variables which had evidence of association (*p* < 0.05) in the crude analyses. Analyses were conducted in Stata 13 using the svy command to adjust for survey design and sampling weights.

## Results

Background characteristics of the sub-sample of women who reported being sexually active are presented in Table 1. Thirty four percent of women had no education and 70% were from rural areas. The large majority were ever married (94%). Over 1 in 4 (28%) reported ever experiencing any form of intimate partner violence.

**Table 1:** Background characteristics of adult women who reported being sexually active, NFHS-4

	NFHS 4 (*N* = 91,818)	
	n*	%
**Age group**		
15–25	21,038	22.9
25–35	34,531	37.6
35 & above	36,249	39.5
**Years of schooling**		
No education	30,939	33.7
1–8 years	28,168	30.7
8+ years	32,711	35.6
**Residence**		
Urban	27,103	29.5
Rural	64,715	70.5
**Wealth Index**		
Poor	36,020	39.2
Middle	19,389	21.1
Rich	36,409	39.7
**Religion**		
Muslim	12,581	13.7
Hindu	69,156	75.3
Others	10,081	11.0
**Caste**		
ST/SC	32,365	35.2
OBC	36,113	39.3
Others	23,340	25.4
**Marital Status**		
Unmarried	5909	6.4
Ever Married	85,909	93.6
**Occupation**		
Not working	62,107	68.4
Working	28,656	31.6
**Distance to health facility**		
No problem	32,874	35.8
Problem	58,944	64.2
**Role in decision making**		
No	30,852	35.9
Yes	55,057	64.1
**Freedom of Mobility**		
No	51,856	56.5
Yes	39,962	43.5
**Ever experienced violence**		
No	47,050	72.0
Yes	18,302	28.0

^*^n are unweighted totals. Information was missing for occupation in 1055 cases, for role in decision-making in 5909 cases and for ever experienced violence in 26,466 cases

An estimated 11.3% of 91,818 ever sexually active women aged 15–49 reported symptoms of RTIs. Of symptomatic women, 39.2% (95% CI: 37.8,40.7) sought any treatment/advice in 2015–16 (Table 2). Of those who reported symptoms (2015–16), 17.0% utilized public services, 22.4% private services, and 2.2% sought treatment in other facilities which included home treatment, NGO/trust providers and community-based services. The overall proportion of women who sought treatment has not changed since the NFHS-3 in 2005–6 (40.4%). The proportion of women who sought care in public facilities increased from 11.4% (CI: 10.5, 12.3) in NFHS-3 to 17.0% (CI: 15.9,18.2) in NFHS-4 (Table 2).

**Table 2:** Proportion of women who reported symptoms of a reproductive tract infection^a^ and sought treatment, NFHS-3 and NFHS-4

	NFHS 3		NFHS 4	
	%	95% CI	%	95% CI
Reported symptoms	11.2	(10.7,11.8)	11.3	(10.9,11.7)
Sought treatment (of symptomatic women)	40.4	(38.9,41.8)	39.2	(37.8,40.7)
Type of facility visited				
Public facility	11.4	(10.5,12.3)	17.0	(15.9, 18.2)
Private facility	24.1	(22.9, 25.4)	22.4	(21.2, 23.7)
Others	7.4	(6.6,8.1)	2.2	(1.8,2.6)

^a^Reported symptoms of RTI include if the women experienced any one of the following: any infection due to sexual contact, genital sore/ulcer; or abnormal genital discharge

**Fig. 1:**
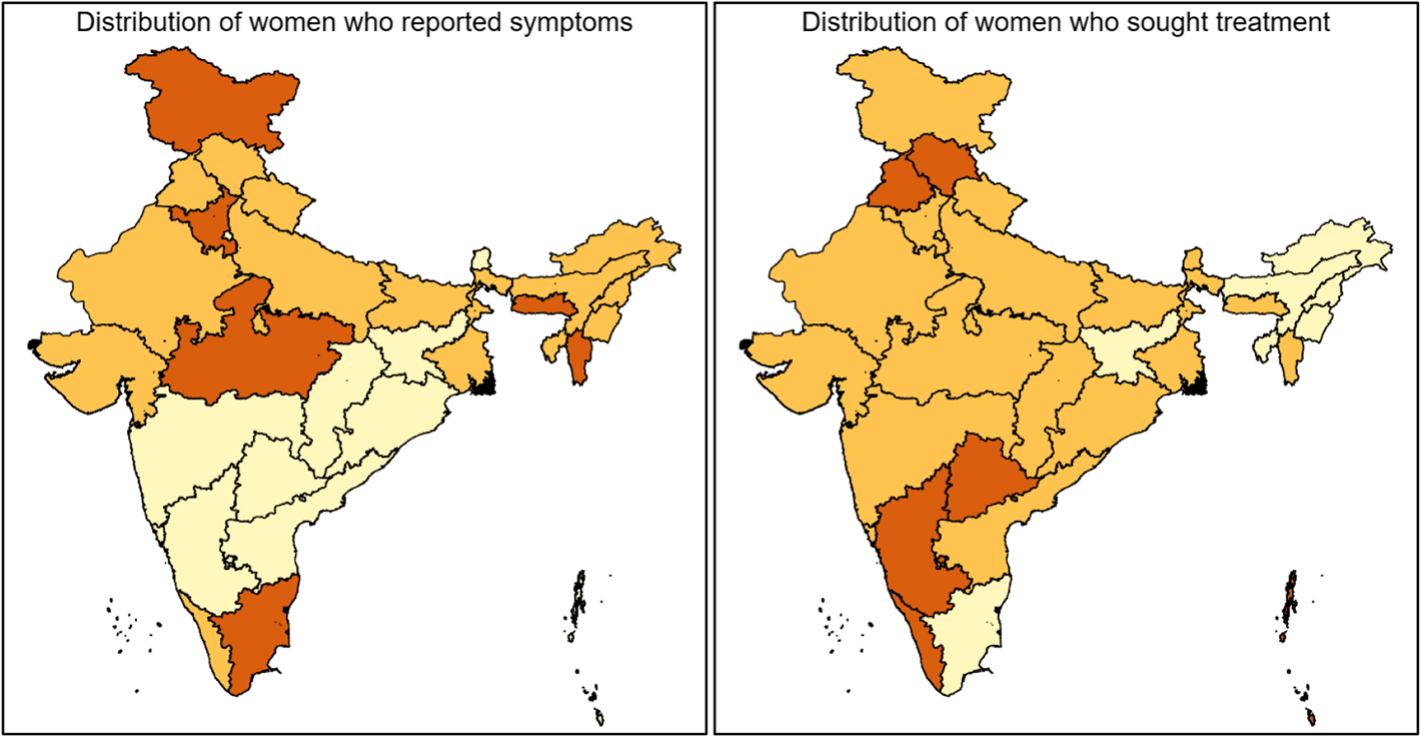
Distribution of women who reported symptoms and of who sought treatment. The maps depicted in Fig. 1 were made by the authors using an India country shape file and STATA 13 (map not to scale, image of Indian state borders as published in Administrative Atlas of India, Census of India 2011). It was digitized in ArcGIS software to generate a country shape file. Distribution of women who reported symptoms  0–6.9  7.0–10.9  11.0–19.0. Distribution of women who sought treatment  0–29.9 30.0–44.9  45.0–65.0.

The proportion of women who reported symptoms and seeking treatment varied considerably across India (Fig. 1). Relatively higher proportions were reported in Meghalaya (26.2%); Haryana (23.4%); Jammu & Kashmir (23.1%), and Mizoram (11.2%). The distribution of seeking treatment, however, did not follow a similar pattern. Treatment-seeking was as low as 7.6% in Nagaland and 19.3% in Assam. Women reported higher levels of seeking treatment in the following states: Punjab (63.9%), Kerala (63.4%), Himachal Pradesh (48.2%), Telangana (47.3%) and Haryana (44.6%).

**Fig. 2:**
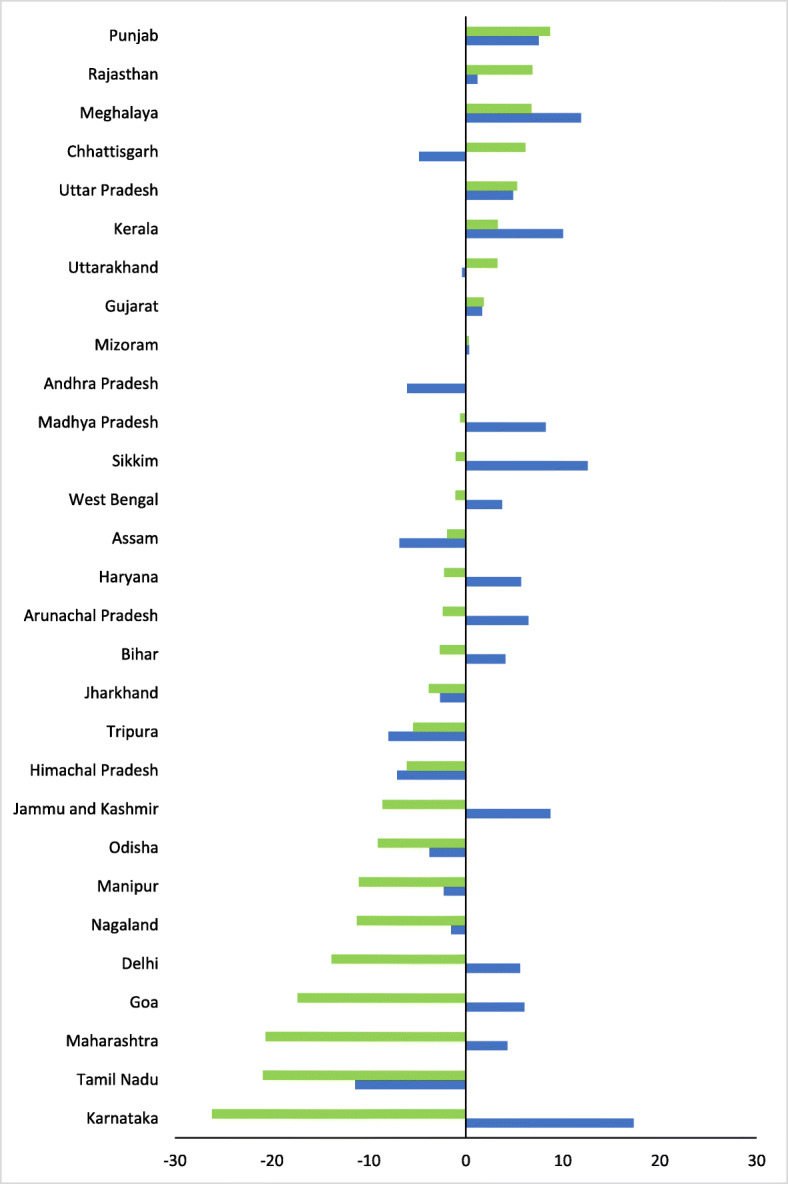
Changes in proportion of symptomatic women who sought treatment by sector, NFHS-3 to NFHS-4 Fig. 2 Private  Public.

Use of public and private services also varied considerably between states (Table 3). A high proportion of symptomatic women accessed public facilities in states such as Karnataka (40.9%), Sikkim (33.4%), Himachal Pradesh (32.9%), Kerala (32.3%) and Jammu and Kashmir (27.1%). This proportion was considerably lower in Jharkhand (2.6%), Nagaland (3.9%), Assam (6.0%) and Bihar (8.0%). Between NFHS rounds 3 and 4, women’s utilisation of private facilities decreased by 1.7% points and increased for public facilities by 5.6% points at the national level, with variation by states (Fig. 2). Increases in use of the public sector were relatively higher in Karnataka (17.3%), Sikkim (12.6%), Meghalaya (11.9%) and Kerala (10.1%), while private utilisation increased in Punjab (8.7%), Rajasthan (6.9%) and Meghalaya (6.8%).

**Table 3:** Proportion of women who sought treatment, by sector, NFHS-3 and NFHS-4 (%)

	Public	Private	Other	Public	Private	Other
	**NFHS 3**			**NFHS 4**		
Nagaland	5.4	15.0	0.0	3.9	3.7	0.0
Arunachal Pradesh	13.1	7.5	1.8	19.6	5.1	0.1
Sikkim	20.8	8.4	0.0	33.4	7.4	0.0
Odisha	21.9	17.6	10.2	18.1	8.5	4.1
Karnataka	23.6	36.3	1.5	40.9	10.1	0.3
Tamil Nadu	32.1	31.7	0.6	20.6	10.8	0.4
Assam	12.9	12.9	15.1	6.0	10.9	2.5
Mizoram	24.0	10.7	0.7	24.3	11.0	1.3
Tripura	17.6	17.6	13.5	9.6	12.2	0.0
Manipur	18.2	24.2	2.5	15.9	13.2	3.2
Jammu and Kashmir	18.4	23.1	3.5	27.1	14.5	0.9
Himachal Pradesh	40.0	22.9	1.6	32.9	16.8	1.1
Rajasthan	17.6	11.3	8.2	18.8	18.2	1.8
Meghalaya	12.5	12.1	0.0	24.3	18.9	0.0
Jharkhand	5.3	22.9	13.6	2.6	19.1	6.2
Madhya Pradesh	9.6	21.2	2.8	17.8	20.6	1.6
Goa	12.1	38.0	2.2	18.1	20.6	4.8
Delhi	15.6	35.5	3.7	21.2	21.7	0.0
Maharashtra	12.8	42.5	1.0	17.1	21.8	0.0
Bihar	3.9	26.0	7.3	8.0	23.4	1.0
West Bengal	9.9	25.1	15.4	13.6	24.1	3.0
Uttarakhand	20.7	21.0	5.2	20.4	24.3	0.7
Haryana	18.7	26.8	12.9	24.4	24.6	0.5
Chhattisgarh	16.3	18.5	4.3	11.5	24.7	1.9
Andhra Pradesh	16.8	26.6	1.0	10.8	26.7	3.3
Gujarat	8.8	27.3	2.2	10.5	29.2	4.4
Uttar Pradesh	7.4	24.9	6.7	12.3	30.2	4.6
Kerala	22.3	29.2	8.0	32.3	32.6	0.3
Punjab	17.1	38.8	10.8	24.6	47.4	1.4
India	11.4	24.1	7.4	17.0	22.4	2.2

Note: proportions are of women who reported symptoms

Table 4 reports both prevalence and treatment-seeking patterns from NFHS-4. Estimates of prevalence and treatment-seeking were lowest amongst younger women. Women with no education and lower economic status reported symptoms in higher proportions but reported relatively lower treatment-seeking. Women who reported distance as a barrier in seeking health services also reported lower treatment-seeking. A higher proportion of women who had ever experienced violence reported symptoms compared to women who did not report exposure to violence, but treatment-seeking was similar in both groups.

**Table 4:** Proportion of women who reported symptoms and sought treatment, by background characteristics, NFHS-4

	n*	Reported symptoms	Sought treatment of those who reported symptoms
**Age group**			
15–25	21,038	10.7(10.0,11.4)	35.09(32.4,37.9)
25–35	34,531	12.4(11.8,13.0)	42.22(40.1,44.4)
35 & above	36,249	10.5(10.0,11.1)	38.39(36.2,40.5)
**Years of schooling**			
No education	30,939	11.8(11.2,12.3)	33.45(31.5,35.5)
1–8 years	28,168	11.7(11.1,12.3)	42.01(39.7,44.4)
8+ years	32,711	10.5(9.9,11.1)	42.3(39.7,44.9)
**Residence**			
Urban	27,103	10.5(9.7,11.3)	41.79(38.8,44.9)
Rural	64,715	11.7(11.2,12.1)	37.99(36.5,39.5)
**Wealth Index**			
Poor	36,020	11.9(11.3,12.5)	32.51(30.6,34.5)
Middle	19,389	11.2(10.5,11.9)	41.05(38.2,44.0)
Rich	36,409	10.7(10.1,11.3)	44.45(42.1,46.9)
**Religion**			
Muslim	12,581	13.0(12.0,14.1)	42.35(38.9,45.9)
Hindu	69,156	10.9(10.5,11.4)	38.21(36.6,39.8)
Others	10,081	12.0(10.7,13.4)	44.85(39.5,50.3)
**Caste**			
ST/SC	32,365	11.7(11.0,12.6)	36.69(34.4,39.1)
OBC	36,113	11.0(10.4,11.5)	38.09(36.1,40.2)
Others	23,340	11.0(10.2,11.8)	43.94(40.8,47.1)
**Marital Status**			
Unmarried	5909	10.4(9.2,11.8)	41.7(36.4,47.2)
Ever Married	85,909	11.3(11.0,11.7)	39.1(37.6,40.6)
**Occupation**			
Not working	62,107	10.7(10.2,11.1)	37.7(36.0,39.5)
Working	28,656	12.4(11.8,13.1)	41.6(39.5,43.9)
**Distance to health Facility**			
No problem	32,874	9.2(8.8,9.7)	42.8(40.2,45.3)
Problem	58,944	16.0(15.2,16.8)	37.5(35.9,39.2)
**Role in decision making**			
No	51,856	9.8(9.2,10.3)	37.5(35.3,39.7)
Yes	39,962	12.2(11.7,12.7)	40.2(38.2,42.2)
**Freedom of Mobility**			
No	30,852	12.8(12.2,13.4)	38.9(37.1,40.7)
Yes	55,057	10.5(10.0,10.9)	39.7(37.7,41.8)
**Ever experienced violence**			
No	47,050	11.7(11.2,12.2)	38.7(36.6,40.8)
Yes	18,302	10.8(10.2,11.3)	38.4(36.1,40.8)

^*^n is the unweighted sample of women who reported being sexually active. Information was missing for occupation in 1,055 cases, for role in decision-making in 5,909 cases and for ever experienced violence in 26,466 cases

Unadjusted odds ratios (Table 5) suggested that age, years of schooling, urban/rural residence, current employment, wealth, and caste were associated with women’s treatment-seeking. There was no evidence that women’s marital status, distance from a health facility, decision-making power, freedom of mobility and exposure to intimate partner violence were associated with seeking treatment. Adjusted analyses (Table 5) indicated evidence for associations of age, education, wealth, caste, and work status with seeking treatment amongst adult women. Women in the age group 25–35 years had higher odds of seeking treatment (aOR 1.27, 95% CI: 1.09, 1.47) as compared to both younger (15–25 years) and older (35 years and above) women. Higher education was associated with reporting seeking treatment: those with 1–8 years of schooling had higher adjusted odds (aOR1.39, 95% CI: 1.21, 1.60), compared to women who had never been to school. The odds of seeking treatment increased with increasing wealth terciles, up to 1.53 (95% CI: 1.30, 1.80) in the highest wealth index. There was strong evidence that women who had engaged in work in the last year reported higher odds of seeking treatment/advice (aOR 1.33, 95% CI: 1.17, 1.51).

**Table 5:** Correlates of treatment-seeking amongst women who reported symptoms, NFHS- 4

	Unadjusted			Adjusted		
	uOR	***p***-value	CI	aOR	*p*-value	CI
**Age group**						
15–25	1.00			1.00		
25–35	1.32	0.00	(1.1,1.5)	1.27	0.00	(1.1,1.5)
35+	1.13	0.11	(1.0,1.3)	1.12	0.16	(1.0,1.3)
**Years of schooling**						
No education	1.00			1.00		
1–8 years	1.45	0.00	(1.3,1.7)	1.39	0.00	(1.2,1.6)
8+ years	1.39	0.00	(1.2,1.6)	1.23	0.01	(1.1,1.4)
**Residence**						
Urban	1.00			1.00		
Rural	0.87	0.06	(0.8,1.0)	1.02	0.79	(0.9,1.2)
**Wealth Index**						
Poor	1.00			1.00		
Middle	1.46	0.00	(1.3,1.7)	1.39	0.00	(1.2,1.6)
Rich	1.58	0.00	(1.4,1.8)	1.53	0.00	(1.3,1.8)
**Caste**						
ST/SC	1.00			1.00		
OBC	1.07	0.32	(0.9,1.2)	0.99	0.88	(0.8,1.1)
Others	1.23	0.01	(1.0,1.3)	1.08	0.38	(0.9,1.3)
**Religion**						
Muslim	1.00					
Hindu	0.86	0.08	(0.7,1.0)			
Others	0.82	0.26	(0.6,1.2)			
**Occupation**						
Not working	1.00			1.00		
Working	1.22	0.00	(1.1,1.4)	1.33	0.00	(1.2,1.5)
**Marital Status**						
Unmarried	1.00					
Ever Married	0.91		(0.71,1.17)			
**Distance to health Facility**						
No problem	1.00			1.00		
Problem	0.89	0.06	(0.8,1.0)			
**Role in decision making**						
No	1.00					
Yes	1.12	0.09	(1.0,1.3)			
**Freedom of Mobility**						
No	1.00					
Yes	1.07	0.25	(1.0,1.2)			
**Ever experienced violence**						
No	1.00					
Yes	1.04	0.56	(0.9,1.2)			

## Discussion

This paper presents findings on women’s treatment seeking for RTIs, which to our knowledge is only the fourth published analysis of large-scale survey data on this issue in India in the past thirty years [21–23]. Our analysis of women’s utilisation of services, across states and over time, suggests that utilisation of services for gynaecological morbidity remains a challenge in most parts of India. Less than 40% of women in India who reported symptoms of RTIs reported seeking care— no improvement since the NFHS-3 ten years earlier. Neighbouring countries such as Nepal and Bangladesh report higher proportions of women who sought treatment for similar symptoms: 48 and 60%, respectively [24, 25]. Our analysis, similar to previous NFHS and DLHS rounds, indicated wide variation in treatment utilisation across Indian states, ranging from 64% in Punjab to 8% in Nagaland. Given that health is a state subject in India, differences in state-level health systems likely contribute to this variation. We could not identify any regional patterns or variation consistent with national rankings of state health system performance [26]. The use of public services increased slightly in the past ten years, with declines in utilisation of private facilities in some states. In Punjab, Rajasthan, Uttar Pradesh and Kerala, utilisation increased in both sectors.

### Barriers to treatment

Our analysis indicates that equitable access to services is of concern: women who are younger, have no education, lower economic status and reside in rural areas reported lower levels of seeking care, similar to findings from a range of community-based studies [17, 18, 27]. In addition, a comparative analysis of NFHS-II, NFHS-III and DLHS RCH-I and II found higher treatment-seeking amongst women with a higher standard of living, education level and age [28]. Similarly, an analysis of NFHS-2 (1998–99) indicated that seeking care varied according to location and by socioeconomic and demographic group: wealthier, older, educated women were more likely to seek treatment [21].

Qualitative research has indicated that individual perceptions—such as a well-established notion of a “culture of silence” around gynaecological ailments—and limited-decision making power prevent treatment-seeking [29–31]. Women’s normalisation of symptoms, or fear/ embarrassment as barriers to treatment, point to deeper-rooted sociocultural ideas around gynaecological morbidity [11, 13, 32]. Women may believe that reproductive health problems, such as vaginal discharge or pain, are simply “women’s fate” and therefore not a condition for which they should seek medical help [15, 33]. For example, a comparison of treatment-seeking for gynaecological, obstetric and contraceptive morbidity in an urban Delhi community noted that a high proportion (92.9%) of women sought care for obstetric morbidity, while only 50.8% of women with gynaecological morbidity sought care [34].

### Health system factors

Community-based research has largely focused on individual or societal barriers to treatment, with relatively less analysis of the availability, acceptability, accessibility, and quality of services in facilities [1, 35]. Available research indicates that women’s perceptions of health system barriers include financial constraints [15, 36, 37], poor perceived quality of care, and limited access to appropriate treatment [14–16]. Studies from Gujarat, West Bengal and Tamil Nadu have highlighted the association between cost of care and treatment seeking for gynaecological morbidity [14–16]. Further, vulnerable populations such as migrants/women with migrant husbands and women in the informal economy may face particular challenges in seeking care [38, 39].

Providers’ knowledge and attitudes towards women’s bodies may also influence women’s utilisation of services. For example, a study amongst private providers revealed that most did not perform internal examinations for women with gynaecological ailments [40]. Male healthcare providers in a rural setting indicated that they were unwilling to examine women’s “private parts” and instead spoke to escorts, rather than women themselves [13]. Lastly, it is possible that the lack of accessible, acceptable treatment may drive over/mis-use of over-the counter medication without adequate care [32].

Although NFHS-4 did not collect data on use of informal providers, earlier rounds of NFHS suggest they were an important source of care. For example, analyses of NFHS-2 indicated that 14% of all consultations for gynaecological symptoms were with informal private providers, with higher use in states such as Bihar (28%), Orissa (25%), West Bengal (39%) and Nagaland (35%). Reported use of informal providers was higher among poorer, lower-caste and uneducated women [21]. More recent community-based studies of women’s preferences also indicate women in rural settings prefer traditional healers, informal providers and home remedies for symptoms of RTIs [14, 41].

Finally, it is noteworthy the NFHS survey rounds straddle the introduction of the National Health Mission, a horizontal health systems reforms largely focused on public sector service delivery related to maternal and child health. Although the program has not appeared to have achieved overall gains in treatment-seeking for RTIs, improvements in states such as Karnataka, Kerala and Himachal Pradesh suggest that the public sector may have become a more viable option for utilisation of gynaecological health services in some states.

### Strengths and limitations

The primary strength of this study is the use of nationally representative data that allows comparison of RTI treatment over time and across states. Building on a strong base of existing community-based literature, we examined a range of potential predictors of women’s treatment-seeking in a nationally representative dataset. However, our analysis was limited by focussing on national estimates. Predictive factors may be context-specific, which may explain wide variation between states or lack of associations between women’s empowerment indicators and treatment-seeking, for example. The NFHS collects data on women’s self-reported symptoms, which could result in underestimates, both due to under-reporting and asymptomatic infections [10, 12]. Lastly, our analysis is limited by using a large national survey that examines a range of health issues, which necessarily has a limited number of questions that can incorporate women’s perceptions or attitudes that influence treatment-seeking decisions [11, 42, 43].

We identified several areas which can be improved in the module on RTIs within the NFHS. The NFHS collects data on symptoms from women who report a history of sexual activity. This criterion excludes women who are not, or choose not to report, being sexually active; morbidity estimates thus do not include reproductive tract infections amongst women who are not sexually active. The long (12-month) recall period for symptoms may limit the reliability of estimates, with potential variation in the population by severity of symptoms as well as socioeconomic status [44]. Our understanding of factors that influenced treatment behaviour could be improved by data on awareness and availability of services, as well as information on the use of informal providers and the reported cure rate.

## Conclusion

Our findings that only two of five women who experienced symptoms of RTIs sought treatment amplifies the need to invest in women’s health in India. While institutional deliveries have doubled between NFHS-3 and NFHS-4 [45] treatment-seeking for RTIs has not changed over two decades—despite national and global priority to expand women’s health services beyond maternal health and family planning. Having achieved impressive gains in the reduction of maternal mortality, India’s policy statements on women’s health are an encouraging commitment. Yet from a health systems perspective, effectively providing services for non-maternal gynaecological issues remains a challenge.

Our findings point to three broad implications for action to improve women’s utilisation of services for gynaecological morbidity. To start, it will be important to estimate gynaecologic morbidity more comprehensively in the NFHS, for example through inclusion of estimates of the well-established burden of menstrual disorders [46, 47]. Further, treatment for women’s gynaecological morbidity should be part of an essential package of services within primary care. Guidelines and training within primary health care and health and wellness centres should ensure inclusion of women’s non-maternal, gynaecologic health needs, particularly symptoms of infections and menstrual disorders along with cancer screening. Lastly, it is critical to continue investments in community-based and government interventions to address gynaecological morbidity, to identify how to bridge treatment-seeking gaps and to inform action at the state and national level.

## Data Availability

The dataset supporting the conclusions of this article is available in the Demographic and Health Surveys (DHS) repository. The data can be downloaded from www.DHSprogram.com.

## Abbreviations

AYUSH: Ayurveda, Yoga & Naturopathy, Unani, Siddha and Homoeopathy
aOR: Adjusted Odds Ratio
CI: Confidence Interval
DLHS: District Level Household Survey
DLHS RCH: District Level Household Survey Reproductive and Child Health
HIV: Human Immunodeficiency Virus
I-WACH: India Strategy for Women’s, Children’s and Adolescents’ Health
NFHS: National Family Health Survey
NGO: Non-Governmental Organisation
OBC: Other Backward Caste
PSU(s): Primary Sampling Units
RTI: Reproductive Tract Infection(s)
SC: Scheduled Caste
SRH: Sexual and Reproductive Health
STI: Sexually Transmitted Infection(s)
ST: Scheduled Tribe
UHC: Universal Health Coverage
